# Perceived Effectiveness of Workplace Violence Prevention Strategies Among Bulgarian Healthcare Professionals: A Cross-Sectional Survey †

**DOI:** 10.3390/healthcare14020220

**Published:** 2026-01-15

**Authors:** Nikolina Radeva, Maria Rohova, Anzhela Bakhova, Sirma Draganova, Atanas Zanev

**Affiliations:** 1 Department of Disaster Medicine and Maritime Medicine, Medical University of Varna, 9002 Varna, Bulgaria; sirmadraganova@gmail.com; 2 Department of Healthcare Economics and Management, Medical University of Varna, 9002 Varna, Bulgaria; mariarohova@abv.bg; 3 Department of Social Medicine and Health Care Organization, Medical University of Varna, 9002 Varna, Bulgaria; anzhela.bakhova@mu-varna.bg; 4 Department of Anaesthesiology, Emergency, Intensive Medicine, Medical University of Varna, 9002 Varna, Bulgaria; naskozanev@gmail.com

**Keywords:** workplace violence, healthcare professionals, prevention strategies, Bulgaria, occupational safety, policy interventions

## Abstract

**Background:** Workplace violence (WPV) is a pervasive occupational hazard in healthcare that undermines staff safety and quality of care. In Bulgaria, WPV remains widespread and underreported, despite recent legislative initiatives. This study assessed healthcare professionals’ perceptions of the effectiveness of WPV prevention strategies and examined how prior exposure shapes these perceptions. **Methods:** A nationwide cross-sectional online survey was conducted in December 2024 with 944 healthcare professionals from multiple sectors. Participants rated the perceived effectiveness of 11 prevention strategies, including environmental/security measures, organizational, and national-level interventions, on a three-point scale. Friedman ANOVA with Kendall’s W assessed overall strategy rankings, while Mann–Whitney U tests with rank-biserial correlations compared specific effectiveness ratings between subgroups defined by WPV exposure (experienced or witnessed vs. not exposed in the previous 12 months). **Results:** In the previous 12 months, 34.7% of respondents reported direct WPV, and 43.4% had either experienced or witnessed incidents. Friedman ANOVA indicated significant differences in perceived effectiveness across strategies (Kendall’s W = 0.13), with stronger differentiation among violence-exposed respondents (W = 0.37) than among non-exposed respondents (W = 0.09). National-level interventions and security/response measures were consistently ranked the highest. Mann–Whitney tests showed significantly higher endorsement of most strategies among violence-exposed professionals, with large effect sizes for security measures and enforcement of sanctions. **Conclusions:** Bulgarian healthcare professionals view WPV prevention as requiring a multicomponent approach that integrates robust national policy with organizational and environmental measures. Direct exposure to violence is associated with stronger support for security-focused and national interventions. These findings inform context-specific, evidence-based WPV prevention programs for Bulgarian healthcare facilities.

## 1. Introduction

Ensuring safety and security within healthcare environments is essential for delivering effective and compassionate patient care, safeguarding healthcare personnel, and promoting the well-being of patients, their families, and the surrounding communities [1]. Staff engagement and a robust safety culture are significantly associated with improved patient outcomes, underscoring that healthcare worker safety and patient safety are fundamentally interconnected [2]. The healthcare environment, characterized by high mobility, substantial emotional demands, and complex social interactions, is widely recognized as a high-risk setting for workplace violence (WPV) [3]. Healthcare professionals are more susceptible to experiencing non-fatal WPV than individuals in other occupational fields [4], making WPV a prevalent occupational hazard and a significant public health concern [5]. This issue extends across Europe, where WPV against healthcare workers is recognized as an occupational hazard with increasing prevalence across multiple health systems. A scoping review conducted in Italy documents violence against hospital-based healthcare workers as an increasing challenge [6].

Workplace violence manifests in various forms, including incivility, verbal abuse, physical threats, and outright physical aggression [7]. Its repercussions are substantial, resulting in adverse physical and psychological health outcomes among exposed healthcare professionals, contributing to unhealthy work environments, and imposing significant financial burdens on healthcare organizations through reduced productivity [8]. A persistent barrier to effective prevention is the chronic underreporting of violent incidents. A systematic review conducted in Spain reports an increasing trend in violence episodes that are frequently inadequately documented and unrecorded [9]. This phenomenon is often attributable to perceptual habituation among healthcare professionals and the absence of clear and accessible reporting mechanisms, both of which substantially impede the development of effective preventive strategies [10]. At the system level, WPV compromises patient safety and quality of care, as affected healthcare workers may provide suboptimal care and demonstrate an increased likelihood of clinical errors [11].

Given the profound and multifaceted impact of WPV, there is an imperative need for robust, evidence-based preventive strategies to safeguard healthcare workers and ensure high-quality care [12]. Such strategies should employ integrated approaches that combine comprehensive training, mental health support, strengthened security measures, and an organizational culture that prioritizes safety and respect [13]. Evidence indicates that multicomponent interventions incorporating environmental measures (e.g., panic buttons, security locks), administrative approaches (e.g., workplace violence policies, safety procedures), and behavioral elements (e.g., staff training) are the most effective [14]. In contrast, single-component interventions, such as training alone, demonstrate limited effectiveness, whereas multicomponent approaches involving stakeholder participation have been shown to significantly reduce violence rates, with studies reporting decreases in assault incidence following implementation [14,15]. Despite this growing body of evidence, a critical gap remains: comparative evidence on the effectiveness of specific prevention components across different healthcare contexts is limited, and healthcare professionals’ perceptions of which interventions are most effective remain largely unexplored, particularly in resource-limited settings [16].

Although WPV in healthcare has been extensively documented worldwide, evidence from Bulgaria indicates a comparable and equally concerning situation. A case study conducted in Bulgaria in 2003 within the Joint Programme on Workplace Violence in the Health Sector reported that 75.8% of healthcare workers had experienced physical or psychological violence [17]. More than two decades later, WPV in Bulgarian healthcare settings remains widespread and substantially underreported, limiting the evidence base for the development of effective prevention strategies. A survey of 254 healthcare workers across four hospitals found that 58% had experienced physical or verbal aggression, yet 91.2% did not report incidents to the police; furthermore, since 2014, more than 600 documented attacks have targeted doctors, nurses, and ambulance personnel [18]. Most recently, surveys conducted in 2025 by the Trend Research Agency for the Bulgarian Medical Association indicated that 56% of physicians had experienced verbal aggression and 7% had been subjected to physical violence. Notably, 41% of respondents attributed responsibility for such aggression to the physicians themselves, reflecting a problematic social perception that undermines recognition of WPV as a legitimate occupational hazard requiring systematic prevention [19].

Bulgaria’s health system is characterized by substantial institutional autonomy and significant private sector participation [20]. The outpatient care sector comprises independent general practitioners and specialists operating self-owned practices, creating isolated workplace environments distinct from hospital settings. Outpatient medical centers and hospitals operate as autonomous entities with independent management authority, allowing considerable discretion in resource allocation and staffing decisions. This fragmented organizational landscape creates variability in workplace safety protocols and violence prevention implementation across facilities, challenging standardized, system-wide approaches. Additionally, critical shortages, particularly among nurses and general practitioners [21], exacerbate workplace violence risk by creating overcrowded facilities, personnel overload, and deteriorating working conditions.

Recently, Bulgaria has implemented various prevention measures, including legislative amendments increasing penalties for assault on medical staff, mandatory training programs for violence recognition, and security infrastructure such as panic buttons and surveillance systems in emergency departments [22]. Despite these efforts, the persistence of WPV and chronic underreporting indicate that existing strategies require systematic evaluation and optimization across healthcare settings. To guide the examination of how violence exposure influences perceptions of prevention strategy effectiveness, the social–ecological model (SEM) [23] was employed as an increasingly applied framework to healthcare violence prevention. This framework suggests that exposure to workplace violence may shift healthcare professionals’ risk perception toward structural interventions, a premise this study systematically tests by comparing strategy endorsement between violence-exposed and non-exposed professionals [24]. Therefore, this study aimed to assess healthcare professionals’ perceptions of the effectiveness of workplace violence prevention strategies in Bulgaria and to examine whether the exposure to workplace violence influences these perceptions. The research question was whether exposure to workplace violence modifies the perceived effectiveness of prevention strategies across environmental, organizational, and national-level interventions. It was hypothesized that healthcare professionals exposed to workplace violence would demonstrate a stronger preference for national-level policy interventions and security measures, whereas non-exposed professionals would place greater emphasis on the development of organizational safety culture, with violence exposure acting as a significant effect modifier of strategy rankings. This study targets a critical knowledge deficit by providing the first systematic analysis in Bulgaria of how exposure to workplace violence shapes healthcare professionals’ prioritization of prevention strategies across intervention levels. Its originality lies in empirically testing the social–ecological model’s prediction that violence exposure acts as a significant effect modifier, fundamentally reorganizing strategy preferences rather than uniformly increasing support, and quantifying these differences with robust effect sizes to inform whether prevention programs should be differentiated by exposure history. The findings provide context-specific evidence to inform targeted intervention design and policy recommendations for the Bulgarian healthcare system.

## 2. Materials and Methods

### 2.1. Study Design and Sample Size

This cross-sectional survey was conducted in December 2024 by a polling agency in Bulgaria, focusing on the experience and perception of healthcare professionals regarding WPV and prevention strategies.

The target population comprised healthcare professionals employed in Bulgarian healthcare facilities across multiple sectors and professional roles. Healthcare professions included physicians, dentists, nurses, midwives, laboratory technicians, imaging technicians, physiotherapists, physician assistants, dental assistants, pharmacists, and healthcare management and administrative support staff. The sample frame consisted of registered members of professional associations across healthcare subsectors: outpatient care, inpatient care, dental care, emergency care, and pharmaceutical care. Participants were included if they were actively employed in a healthcare facility within the 12 months prior to the survey, either as employees or as self-employed practitioners operating private practices in Bulgaria. Participants were excluded if they had not practiced their profession within the previous 12 months or were employed in healthcare positions abroad.

Sample size calculation was conducted using G*Power software version 3.1.9.7 to estimate the minimum sample size required to detect significant differences between groups in perceived effectiveness ratings. The calculation was based on comparing healthcare professionals’ perceptions between those with and without workplace violence exposure. A priori power analysis was conducted using a point-biserial correlation model. Using Cohen’s effect size conventions [25], we specified a medium effect size (*r* = 0.30), a statistical power of 0.95, and a two-tailed significance level of α = 0.05. This calculation indicated a minimum required sample size of 132 participants. Given the ordinal nature of the data and the need for robust analysis, it was decided to aim for approximately 150 participants.

Additionally, for examining differences in effectiveness perceptions across 11 prevention strategies within each exposure group, a Friedman ANOVA test was planned. Sample size requirements for this nonparametric repeated-measures analysis were calculated using the repeated measures ANOVA function in G*Power with a medium effect size (f = 0.25), statistical power of 0.95, and 11 measurements. Following Lehmann’s recommendations for adjusting parametric sample sizes to nonparametric tests [26], a 15% adjustment was applied, yielding a minimum of approximately 25 participants per group.

The achieved sample of 944 healthcare professionals across all professional categories and facility types substantially exceeded both the point-biserial calculation requirement (150 participants) and the Friedman ANOVA requirement (25 participants), providing exceptional statistical power to detect meaningful effects, conduct robust stratified analyses, and assess effect modification by violence exposure.

Eligible healthcare professionals were selected through random sampling from registered members of professional associations. From this sampling frame, 1700 individuals were initially selected, assuming an expected response rate of 45–50% to achieve the required sample size. An invitation email containing a brief study description and a link to the online questionnaire was sent to all selected healthcare professionals, and one reminder was distributed two weeks later. A total of 944 individuals completed the survey, yielding a response rate of approximately 55%. The final sample included healthcare professionals from diverse roles and healthcare settings, and this sample size was considered sufficient for the planned statistical analyses.

### 2.2. Data Collection Instrument

The data collection instrument was a structured questionnaire designed to capture demographic and professional characteristics, workplace violence exposure experiences, and healthcare professionals’ perceptions of effectiveness for workplace violence prevention strategies. The demographic and professional characteristics section included items such as age, gender, professional role, years of professional experience, and type of healthcare facility. The workplace violence exposure section assessed experiences in the past 12 months with items such as: ‘Have you personally experienced physical aggression in the workplace?’, ‘Have you personally experienced verbal aggression (e.g., insults, threats, humiliation)?’, and ‘Have you personally experienced sexual harassment in the workplace?’ The questionnaire included a list of potential workplace violence prevention strategies, which were informed by the World Health Organization (WHO) and International Labor Organization (ILO) project conducted in Bulgaria [17].

The initial catalogue of 12 strategies underwent content refinement through expert consultation. A panel of five experts in public health and health policy independently assessed each item for clarity and relevance using a 4-point rating scale. For each strategy, only items that were rated as 3 or 4 (i.e., relevant) by the panel were retained in the final catalogue, and based on the experts’ written comments, four items were further revised to improve clarity and ensure relevance to the current context of the Bulgarian health system. The finalized list consisted of 11 strategies grouped into three domains: environmental control and security measures (4 items), organizational interventions (4 items), and national-level interventions (3 items).

A pilot study with 80 healthcare professionals, who were not included in the main study sample, was conducted prior to full-scale administration to evaluate questionnaire clarity, relevance, and feasibility. Participants completed a paper-based version of the questionnaire and provided feedback on item clarity, the perceived relevance of each strategy, and the comprehensiveness of the prevention strategy list. Overall, items were rated as clear and relevant, and participants expressed a preference for a simpler response scale when rating the strategies. Accordingly, minor wording changes were made and a simplified rating scale was adopted to improve clarity and ease of completion, while ensuring that the questionnaire accurately captured the intended information. Data from the pilot study were used solely for refinement of the questionnaire and were not included in the final analysis.

Perceived effectiveness of prevention strategies was assessed using a 3-point rating scale: 1 = “Low effectiveness”; 2 = “Moderate effectiveness”; 3 = “Very high effectiveness”. All response categories were labeled. This scale format was chosen to provide consistency with pilot participant preferences for reduced completion burden.

### 2.3. Statistical Analyses

Study Variables. The primary outcome was perceived effectiveness of workplace violence prevention strategies, measured on a 3-point ordinal scale for each of the 11 strategies. The primary predictor variable was workplace violence exposure status in the 12 months preceding the survey, categorized as: (1) exposed—healthcare professionals who reported experiencing or witnessing at least one incident of workplace violence (physical aggression, verbal aggression, or sexual harassment); (2) unexposed—healthcare professionals who reported no such experiences. The exposed group comprised 410 participants (43.4% of total sample), and the unexposed group comprised 534 participants (56.6%). These subsample sizes exceed minimum requirements for the planned nonparametric comparative analyses.

Data Analysis. Descriptive analysis characterized the total sample. Normality of effectiveness ratings was assessed using the Shapiro–Wilk test across all 11 strategies. Significant departures from normality (*p* < 0.001) and the ordinal nature of the 3-point effectiveness scale justified the use of nonparametric methods. The Friedman ANOVA was selected as the primary test to compare perceived effectiveness ratings across the 11 strategies, conducted at three analytical levels: (1) the entire sample (N = 944); (2) the violence-exposed subsample (n_1_ = 410); and (3) the unexposed subsample (n_2_ = 534). This stratified approach examined whether the ordering of strategy effectiveness differed as a function of violence exposure history. Kendall’s W coefficient of concordance was calculated as an effect size to quantify the magnitude of differences in effectiveness ratings across the 11 prevention strategies. When omnibus Friedman tests yielded significant results (*p* < 0.001), post hoc pairwise comparisons were conducted using the Wilcoxon signed-rank test with Holm adjustment to identify specific differences in strategy effectiveness rankings [27]. The results from the overall sample were compared with those from the two subsamples to explore any variations in perceptions based on experiences with workplace violence. To determine whether violence exposure status modified perceived effectiveness of individual prevention strategies, Mann–Whitney U tests were conducted to compare effectiveness ratings for each strategy between the violence-exposed and unexposed groups, with rank-biserial correlation coefficients calculated as measures of effect size to quantify the magnitude of differences between groups.

A significance level of *p* < 0.05 was established, and effect sizes were calculated for statistically significant findings. Statistical analyses were performed using R Statistical Software version 4.5.2.

### 2.4. Ethical Considerations

This study received ethical approval from the Medical University of Varna Research Ethics Committee (Ref. No. 4/12.04.2024). The distribution of the questionnaire was managed by the polling agency, and no personal identifying information was collected from participants, ensuring confidentiality and anonymity. Participants provided informed consent via the online survey platform following presentation of an information sheet describing study purpose, voluntary nature, right to withdraw, and data protection procedures.

## 3. Results

### 3.1. Sample Characteristics

Demographic and professional characteristics of the sample are presented in Table 1. The majority of respondents were in the 55–64 age group (Table 1), reflecting the aging workforce within the Bulgarian healthcare system. The gender distribution showed a predominance of males. Regarding the place of residence, most respondents lived in district cities (44.6%), followed by the capital (40.3%), which corresponded to the concentration of healthcare facilities in major urban areas.

The professional composition of the sample revealed a strong representation of physicians and nursing professionals, together accounting for more than 71% of participants. These groups are typically at the forefront of patient care and therefore may be more exposed to incidents of workplace violence. Most respondents reported extensive professional experience of over 20 years (Table 1). The sample encompassed a range of healthcare settings, with hospitals and outpatient care facilities most frequently represented. The acute care demands in hospitals may influence both the prevalence and types of violence encountered.

A considerable proportion of healthcare professionals reported experiencing violence within the past 12 months. Specifically, 34.7% of respondents (95% CI: 31.7–37.8%) reported personally experiencing aggression. Physical aggression was reported by 6.8% (95% CI: 5.3–8.6%), verbal aggression by 32.1% (95% CI: 29.1–35.2%), and sexual harassment by 3.5% (95% CI: 2.4–4.9%) of respondents. In the previous 12 months, 39.0% of physicians and 41.8% of nursing and midwifery professionals experienced workplace violence, representing the most affected professional groups. When stratified by healthcare facility type, hospital employees experienced the highest violence exposure at 53.4%.

Beyond direct victimization, the survey also examined exposure to violent incidents through witnessing aggressive behavior. Of the respondents, 32.0% (95% CI: 29.0–35.1%) reported having witnessed workplace violence events, while 43.4% (95% CI: 40.3–46.6%) reported having been exposed to violence, either personally or through colleagues (Table 1). This findings suggested that violence in healthcare settings extended beyond individual experiences and constituted a pervasive issue affecting the overall work environment.

### 3.2. Within Group Comparison

Healthcare professionals demonstrated statistically significant differences in perceived effectiveness of workplace violence prevention strategies (Friedman ANOVA, *p* < 0.001), with at least one strategy showing notably different median ratings (Table 2). Across the entire sample, national-level interventions received the highest median ratings, while organizational and environmental measures received more moderate endorsement. However, agreement among healthcare professionals regarding strategy rankings was low (Kendall’s W = 0.13), indicating substantial individual variation in perceived effectiveness.

Notably, respondents’ exposure to workplace violence substantially influenced their strategy preferences. Violence-exposed respondents (Group 1) demonstrated significantly stronger differentiation in their ratings compared to unexposed respondents (Group 2), as indicated by Kendall’s W values (Table 2). This pattern may reflect heightened awareness or sensitivity among violence-exposed professionals, who are likely more attuned to the relevance and perceived effectiveness of specific preventive or response measures.

Violence-exposed respondents (Group 1) exhibited a pronounced preference for national-level policy interventions, ranking legislative measures, public awareness campaigns, and enforcement of sanctions as substantially more effective than other strategies (Table 3). These strategies emerged as the top-rated items, suggesting that individuals with direct experience or observation of workplace violence prioritized strong policy interventions aimed at mitigating such incidents. The clear hierarchy observed in the responses underscored a shared recognition of the importance of comprehensive national-level measures to prevent workplace violence.

Unexposed respondents (Group 2) demonstrated more compressed preference ratings, with enforcement of sanctions ranked highest but with smaller differences across items (Table 3). Notably, fostering a safety culture received relatively higher endorsement in Group 2 compared to Group 1. This finding suggests that individuals without direct exposure to violence placed greater emphasis on fostering a positive safety culture as a proactive measure to prevent violence.

Preferences concerning security measures and access control, staffing levels, training, and response protocols to violent incidents were shared between the two groups. While both groups recognized the importance of these strategies, the urgency and prioritization appeared to differ according to their experiences with workplace violence.

The overall ranking of strategies across both groups revealed a blend of the two profiles, suggesting that although respondents broadly agreed on the key domains of effective intervention, the relative emphasis on specific measures varied depending on exposure to workplace violence.

Post hoc pairwise comparisons (Wilcoxon signed-rank tests with Holm adjustment) revealed that the largest differentiation occurred between the highest-ranked strategies (items 8–11) and several lower-ranked items (items 2, 5, 7), confirming meaningful stratification in perceived effectiveness across the prevention strategies (Table 3). Although the ranking pattern demonstrated a consistent and meaningful differentiation in endorsement across the items, the absolute magnitude of these differences remained modest.

### 3.3. Between Group Comparison

Violence-exposed and unexposed respondents differed significantly in their endorsement of most workplace violence prevention strategies (Mann–Whitney U tests; Table 4). The most substantial between-group differences emerged for eight strategies (items 1, 2, 3, 4, 5, 6, 7, and 9), with violence-exposed respondents consistently rating these approaches more favorably than unexposed respondents.

The national-level policy interventions, particularly items 10 and 11 demonstrated a significant between-group differentiation, with medium effect sizes (*r* ≈ 0.46–0.50). Violence-exposed respondents reported higher endorsement of these strategies compared to unexposed respondents, reflecting their recognition of the critical importance of policy-level approaches in preventing workplace violence.

Items 1, 2, 3, 4, 5, 6, and 9 showed even larger effect sizes (*r* ≈ 0.58–0.67), indicating the most pronounced between-group divergence. These findings underscore a consistent pattern: professionals with direct experience of workplace violence expressed significantly greater support for targeted prevention strategies and enhanced security measures than those without such exposure. In contrast, the safety culture strategy (item 8) showed no significant difference between groups, suggesting that both violence-exposed and unexposed respondents perceive this approach as equally important for preventing workplace violence.

Taken together, the items with the largest effects likely represent key priorities that could inform the design and implementation of targeted organizational interventions and policy measures to mitigate workplace violence and its consequences.

The analysis revealed that healthcare professionals’ preferences for workplace violence prevention strategies are substantially shaped by their exposure to violence. Violence-exposed professionals prioritized national-level policy interventions with greater conviction, whereas unexposed professionals distributed their emphasis more evenly across strategies. The findings indicate that while healthcare professionals broadly recognize the importance of comprehensive, multi-level prevention strategies, the urgency and prioritization of specific interventions vary according to direct experience with workplace violence.

## 4. Discussion

In this study, more than one-third of healthcare professionals reported experiencing workplace violence, and nearly half reported having either experienced or witnessed violent incidents, underscoring the pervasive nature of WPV in Bulgarian healthcare settings. A comprehensive, evidence-based approach is essential to effectively reduce WPV against healthcare professionals. The present findings emphasize the importance of integrating national-level and organizational interventions with environmental control and security measures, within a unified prevention strategy that aligns with the preferences and priorities identified among respondents.

The demographic profile of the sample, predominantly older (aged 55–64 years) and male, reflects the composition of Bulgaria’s aging healthcare workforce. Although previous studies indicate that age and gender may influence both exposure to workplace violence and the adoption of prevention strategies [10,24], the primary focus of the present study was effect modification by violence exposure rather than demographic predictors. Our analyses demonstrated that workplace violence exposure is an important determinant of variations in strategy preferences.

The pronounced preference among violence-exposed professionals for national-level policy interventions and security measures, with weak-to-moderate differentiation, aligns with social–ecological theory [29]. The SEM predicts that exposure to violence prompts workers to recognize that individual-level training or even organizational safety culture are insufficient without corresponding interventions at organizational (robust incident reporting systems) and societal levels (legislative enforcement, law enforcement effectiveness) [24]. Large effect sizes for security measures and enforcement of sanctions reflect this shift toward structural accountability [30]. Understanding these perceptions remains crucial for developing targeted interventions that address healthcare workers’ safety concerns and improve organizational responses to WPV [31].

These findings are consistent with previous studies advocating for the establishment of robust policy frameworks and clearer legal protections for healthcare professionals [32]. Legislative action, coupled with consistent enforcement, has been shown to strengthen institutional accountability, clarify consequences for perpetrators, and signal societal recognition of violence against healthcare personnel as an unacceptable norm [33]. Public education campaigns may further reinforce this message by shaping community expectations, improving patient and visitor behavior, and fostering a culture of respect toward healthcare staff [34]. At the organizational level, national policies must be accompanied by internal support systems that translate zero-tolerance standards into routine practice [35].

Organizational interventions and security measures were consistently endorsed by both groups, with respondents emphasizing the importance of clear protocols for responding to violent incidents, structured reporting procedures accompanied by counseling support, and initiatives aimed at strengthening overall safety culture. Notably, participants without prior violence exposure ranked safety culture improvements more highly, whereas violence-exposed respondents demonstrated significantly stronger support for incident response protocols and formal reporting systems. These between-group differences highlight the heightened sense of urgency among violence-exposed professionals and are consistent with recommended practices, including the implementation of dedicated aggression-management teams, standardized reporting mechanisms, and systematic post-incident counseling [10].

Security-focused measures, such as access control, demonstrated large effect sizes favoring violence-exposed respondents, indicating a heightened priority for immediate protection through interventions such as surveillance, screening, and panic alarms in high-risk areas. These preferences are consistent with existing evidence supporting the effectiveness of metal detectors, CCTV, and on-site security personnel in deterring violent incidents and enabling rapid response [8,33]. Environmental adjustments, including patient screening, further complement these measures by reducing potential triggers through improved signage and waiting-area design [36].

Respondents also emphasized increasing staffing levels and improving working conditions as critical components of WPV prevention. These priorities reflect an understanding that operational pressure, such as prolonged waiting times and overcrowding, can intensify tensions and elevate the risk of violent incidents [37]. The emphasis on adequate staffing and equitable workload distribution is consistent with evidence indicating that operational improvements are effective strategies for reducing violence risk in healthcare settings [38]. Previous studies further demonstrate that sufficient staffing and manageable workloads contribute to lower stress levels, improved mental health, and greater job satisfaction among healthcare professionals [39]. These outcomes are not only essential for sustaining a healthy workforce but are also intrinsically linked to the prevention of workplace violence.

Training in communication, de-escalation, and self-defense was recognized by both groups as an important component of workplace violence prevention, although it received a comparatively lower overall ranking. This pattern suggests that such training is perceived less as a primary intervention and more as a complementary skill-building measure that supports, rather than substitutes for, broader organizational changes [33]. The consistent endorsement of a strong safety culture across groups further underscores a shared recognition of the importance of proactive norms and expectations in clinical settings. Importantly, these findings align with recommendations cautioning against rigid or overly forceful responses, instead emphasizing approaches that prevent escalation and prioritize the safety of both staff and patients [40].

The findings of this study have important implications for policymakers and organizational leaders responsible for addressing workplace violence. The pronounced preference among violence-exposed respondents for national-level interventions, particularly legislative measures and the enforcement of sanctions, underscores the need for targeted policy initiatives that incorporate the perspectives and lived experiences of affected healthcare professionals. Furthermore, the emphasis on raising public awareness and strengthening support for zero-tolerance policies indicates that education and broader cultural change are essential components of effective workplace violence prevention strategies.

For healthcare organizations, the findings indicate that fostering a strong safety culture is particularly important, especially for healthcare professionals not exposed to workplace violence. The emphasis placed by this group on safety culture as a preventive measure highlights the value of establishing organizational environments in which employees feel supported and secure, potentially functioning as an upstream intervention that mitigates risk before violent incidents occur.

This study has several important limitations that should be considered when interpreting the findings. First, the cross-sectional design precludes the establishment of causal relationships between workplace violence exposure and perceived effectiveness of prevention strategies. As both exposure and outcome (effectiveness perceptions) were collected simultaneously, temporal sequencing cannot be determined. Second, the study relied exclusively on self-reported data regarding violence exposure in the preceding 12 months and perceived strategy effectiveness. Recall bias may have affected respondents’ recollections of violent incidents, potentially leading to underreporting or misclassification of exposure. Third, the three-point effectiveness rating scale provides limited granularity for distinguishing between nuanced perception differences across prevention strategies. This scale limitation may obscure subtle differences in perceived effectiveness across strategies, particularly among professionals with moderate perceptions. Fourth, although the study achieved comparatively high response rate, the non-response introduces potential selection bias. Non-respondents may systematically differ from respondents with respect to workplace violence exposure or perceptions of prevention strategy effectiveness. Fifth, the online survey administration may have introduced additional response bias, as data collection could influence participation and response patterns. Finally, as the study population was limited to healthcare professionals in Bulgaria, the generalizability of findings to healthcare workers in other countries or to workers in other industrial sectors may be limited. Context-specific and cultural factors should be considered when applying these findings to other populations.

Despite these limitations, the large sample size and stratified analytical approach strengthen the generalizability of findings regarding healthcare professionals’ perceptions of workplace violence prevention strategy effectiveness within the Bulgarian healthcare system. To address these limitations, future research should employ objective incident data from workplace records to corroborate self-reported violence exposure and employ expanded rating scales with greater discriminatory capacity to distinguish nuanced differences in perceived strategy effectiveness.

## 5. Conclusions

This study reveals significant differences in healthcare professionals’ preferences for workplace violence prevention strategies, with pronounced variations between those with direct exposure to violence and those without such experiences. Although the overall differences in strategy rankings are modest in magnitude, they carry important implications for intervention design and implementation. Violence-exposed respondents consistently prioritized national-level policy interventions and security measures, reflecting their recognition of the need for systemic, structural approaches to address violence at an institutional and regulatory level. Those without direct exposure emphasized the development of organizational safety culture, suggesting a preference for preventive approaches.

These divergent preferences underscore the importance of adopting a multicomponent interventions and differentiated approach to violence prevention that acknowledges both the perspectives of affected professionals and the broader organizational context. The findings will contribute to the ongoing discourse on enhancing workplace safety and developing targeted interventions in the healthcare sector.

## Figures and Tables

**Table 1:** Demographic, professional characteristics, and workplace violence exposure of health professionals respondents (N = 944).

Characteristics	Frequency (*n*)	Proportion (%)
**Gender** ^†^		
Male	564	59.7
Female	378	40.1
**Age** (years)		
18–34	26	2.8
35–44	85	9.0
45–54	113	12.0
55–64	610	64.6
65+	110	11.7
**Profession** ^††^		
Physician	333	35.3
Dentist	84	8.9
Nursing and midwifery professional	340	36.0
Health associate professional (laboratory technician, imaging technician)	59	6.3
Other health professional (physiotherapist, physician/dental assistant)	52	5.5
Pharmacist	41	4.3
Health management and support personnel	35	3.7
**Work experience** (years)		
<5	30	3.2
5–10	133	14.1
11–20	199	21.1
>20	582	61.7
**Primary occupation**		
Hospital care	406	43.0
Emergency care	62	6.6
Outpatient care	444	47.0
Pharmaceutical care	19	2.0
Other	13	1.4
**Place of residence**		
Capital	380	40.3
District city	421	44.6
Small town	133	14.1
Village	10	1.1
**Workplace violence exposure in the last 12 months**		
Experienced	328	34.7
Witnessed	302	32.0
Either experienced or witnessed	410	43.4
Without such experience	534	56.6

Note: ^†^ Missing data: 2 (0.2%). ^††^ Profession categories are based on the International Standard Classification of Occupations (ISCO-08) [28].

**Table 2:** Friedman ANOVA test summary results.

	All Respondents	With Violence Exposure (Group 1)	Without Violence Exposure (Group 2)
***n***	944	410	534
Friedman χ^2^ (df = 10)	384.56	371.91	120.82
*p*	<0.001	<0.001	<0.001
Kendall’s W	0.13	0.37	0.09
Magnitude	very weak	weak to moderate	negligible

**Table 3:** Mean ranks and rank positions of prevention strategies based on Friedman test.

No.	Prevention Strategy (Item)	All Respondents		With Violence Exposure (Group 1)		Without Violence Exposure (Group 2)	
		Mean Rank	Rank Position	Mean Rank	Rank Position	Mean Rank	Rank Position
**Environmental control and security measures**							
1	Security measures and access control	6.06	5	6.12	4	6.01	6
2	Patient screening	5.40	10	5.17	11	5.57	9
3	Protocols for responding to violent incidents	6.09	4	6.10	5	6.07	5
4	Reporting procedures, organizational support, and counseling	5.89	7	5.80	6	5.96	7
**Organizational interventions**							
5	Improving working conditions	5.36	11	5.36	9	5.36	11
6	Increasing staffing levels (adequate staffing)	5.65	8	5.39	8	5.84	8
7	Training (communication skills, conflict de-escalation, self-defense)	5.45	9	5.33	10	5.54	10
8	Improving organizational culture (safety culture)	5.94	6	5.54	7	6.25	3
**National-level interventions**							
9	Enhancing the effectiveness of law enforcement and prosecution service	6.84	1	6.95	3	6.76	1
10	Introducing new legislative measures	6.61	3	7.16	1	6.19	4
11	Raising public awareness and support for zero-tolerance policies	6.72	2	7.09	2	6.43	2

Note: Mean rank: the average rank assigned to each item. Rank position: the relative position of each item based on its mean rank, where a lower mean rank indicates a higher-ranked item. Rank 1 represents the highest rank (most preferred).

**Table 4:** Comparison of strategies ratings between groups based on Mann–Whitney U test.

No.	Prevention Strategy (Item)	With Violence Exposure (Median1)	Without Violence Exposure (Median2)	U	*p*	*r*	Magnitude
**Environmental control and security measures**							
1	Security measures and access control	3	2	127,027.0	<0.001	0.61	large
2	Patient screening	2	2	120,965.5	0.003	0.66	large
3	Protocols for responding to violent incidents	3	2	128,529.0	<0.001	0.59	large
4	Reporting procedures, organizational support, and counseling	3	2	125,525.5	<0.001	0.62	large
**Organizational interventions**							
5	Improving working conditions	2	2	124,770.0	<0.001	0.63	large
6	Increasing staffing levels (adequate staffing)	2	2	120,189.0	0.005	0.67	large
7	Training (communication skills, conflict de-escalation, self-defense)	2	2	124,812.0	<0.001	0.63	large
8	Improving organizational culture (safety culture)	2	2	111,499.5	0.591	-	-
**National-level interventions**							
9	Enhancing the effectiveness of law enforcement and prosecution service	3	3	130,048.0	<0.001	0.58	large
10	Introducing new legislative measures	3	2	143,177.5	<0.001	0.46	medium
11	Raising public awareness and support for zero-tolerance policies	3	2	139,007.5	<0.001	0.50	medium

Note: U = Mann–Whitney U statistic; *r*—rank-biserial correlation coefficient: |*r*| ≈ 0.1 is negligible, 0.3 small, 0.5 medium, ≥0.5 large [25].

## Data Availability

The data presented in this study are available on reasonable request from the corresponding author due to ethical restrictions.

## Abbreviations

The following abbreviations are used in this manuscript:

WPV	Workplace violence
SEM	Social–ecological model
